# Advancements in strategies for overcoming the blood–brain barrier to deliver brain-targeted drugs

**DOI:** 10.3389/fnagi.2024.1353003

**Published:** 2024-08-26

**Authors:** Zhichuang Qu, Juan Luo, Zheng Li, Rong Yang, Jiaxi Zhao, Xin Chen, Sixun Yu, Haifeng Shu

**Affiliations:** ^1^Department of Neurosurgery, Meishan City People's Hospital, Meishan, China; ^2^Department of Neurosurgery, General Hospital of Western Theater Command, Chengdu, China; ^3^Department of Neurosurgery, Affiliated Hospital of Southwest Medical University, Luzhou, China; ^4^College of Medicine of Southwest Jiaotong University, Chengdu, China

**Keywords:** blood–brain barrier, brain-targeted drugs, nanomaterials, cell transport, physical stimulation

## Abstract

The blood–brain barrier is known to consist of a variety of cells and complex inter-cellular junctions that protect the vulnerable brain from neurotoxic compounds; however, it also complicates the pharmacological treatment of central nervous system disorders as most drugs are unable to penetrate the blood–brain barrier on the basis of their own structural properties. This dramatically diminished the therapeutic effect of the drug and compromised its biosafety. In response, a number of drugs are often delivered to brain lesions in invasive ways that bypass the obstruction of the blood–brain barrier, such as subdural administration, intrathecal administration, and convection-enhanced delivery. Nevertheless, these intrusive strategies introduce the risk of brain injury, limiting their clinical application. In recent years, the intensive development of nanomaterials science and the interdisciplinary convergence of medical engineering have brought light to the penetration of the blood–brain barrier for brain-targeted drugs. In this paper, we extensively discuss the limitations of the blood–brain barrier on drug delivery and non-invasive brain-targeted strategies such as nanomedicine and blood–brain barrier disruption. In the meantime, we analyze their strengths and limitations and provide outlooks on the further development of brain-targeted drug delivery systems.

## 1 Introduction

Delivery of medicines to the central nervous system (CNS) is restricted by the blood–brain barrier (BBB), which leads to a significant reduction in therapeutic efficacy (Abbott et al., 2010; Abbott, 2013; Daneman and Prat, 2015). The BBB is a micro-vascular network that surrounds the CNS and separates it from the peripheral circulation. This unique physiological structure plays an important role in precisely regulating the transport of bio-molecules between peripheral blood and brain tissues as well as maintaining the homeostasis of the internal environment of the central nervous system. Simultaneously, this barrier also prevents most large molecule drugs and more than 98% of small molecule drugs from entering brain tissue (Daneman and Prat, 2015; Pandit et al., 2020). This barrier consists of endothelial cells (ECs), pericytes (PCs), basement membranes and astrocytes (Ribatti et al., 2006; Abbott, 2013; Daneman and Prat, 2015). Endothelial cells form extensive tight junctions (TJs) through trans-membrane proteins, cytoplasmic attachment proteins, and cytoskeletal proteins, which restrict the cellular transport of molecules in the peripheral circulation and allow passive diffusion of only lipid-soluble drugs with molecular weights of less than 400-600da (Abdullahi et al., 2018; Zhang et al., 2021). Transporters, receptors, and ion channels tightly control the uptake of ions and small molecules, and the net negative charge on the surface of endothelial cells resists molecules with the same charge (Pardridge, 2012, 2016; Dong, 2018; Sweeney et al., 2019). Although trans-cellular vesicles are able to carry many bio-molecules across the BBB by virtue of their phospholipid bilayer structure, high trans-endothelial resistance restricts this pathway (Saint-Pol et al., 2020; Rehman et al., 2023). Simultaneously, the efflux transporter at the blood–brain barrier, p-glycoprotein, actively removes foreign macro-molecules (Obermeier et al., 2013; Arvanitis et al., 2020; Kadry et al., 2020). In addition, a discontinuous layer of PCs surrounds the basal surface of the endothelial cell wall. The ECs and PCs are also covered by a basement membrane that resembles the basement membrane, which is tightly enveloped by the telopods of astrocytes (Liebner et al., 2018). Therefore, it has been a challenge for researchers to overcome the mentioned limitations of pharmaceutical transport and enhance the concentration of drugs in the CNS.

In recent years, the intensive development of nanomaterial science and the cross-fertilization of medical engineering disciplines have opened up unlimited possibilities for regulating the permeability of the blood–brain barrier, involving various processes of drug transport (Dousset et al., 2006; Vellinga et al., 2008; Zhou et al., 2018; Xie et al., 2019; Li T. et al., 2022). Nanomaterials have a promising potential for a wide range of applications in the biomedical field, mainly for medical diagnostics and therapeutics, due to their specific physical, chemical, and biological properties (Sha and Badhulika, 2020; Chen et al., 2021; Liu and Jiang, 2022; Yuan et al., 2023). Medical nanomaterials exhibit unique surface properties, such as enhanced adhesion, activity, and selectivity, making them uniquely suited for drug delivery. Researchers have been able to control their size, shape, and surface chemistry in order to achieve targeted drug delivery *in vivo*, improving drug efficacy and reducing side effects (Khor et al., 2019; Kladko et al., 2021; Collin et al., 2022). They are constructed from many substrates, including inorganic (e.g., metal/metal oxide particles) (Lee et al., 2011; Du et al., 2021; Wang et al., 2021), organic (e.g., liposomes, hydrogels) (Carne et al., 2011; Luo et al., 2019; Niu et al., 2022), and biofilm-derived materials (e.g., exosomes, vesicles, etc.) (Benoit et al., 2019; Qi et al., 2019; Cui et al., 2022). In addition, physical means such as focused ultrasound and surface acoustic waves (SAW) have opened up the possibility of transiently increasing blood–brain barrier permeability and enhancing drug efficacy (Etame et al., 2012; Phenix et al., 2014; Alekou et al., 2021; Hsiao et al., 2023; Qi M. et al., 2023). Furthermore, with the intensive development of physical techniques such as ultrasound, new methods for transiently reversible enhancement of local blood–brain barrier permeability are being explored. Drug-carrying micro-bubbles open the blood–brain barrier under ultrasound stimulation through stable cavitation and inertial cavitation effects, increasing the concentration of pharmaceuticals in the lesion area (Wang et al., 2022). SAW is an acoustic wave that propagates along the surface of a medium, with properties such as concentrated energy and ease of manipulation. Stimulation of SAW alters the distribution of tight junction proteins in neighboring cells and enlarges the cellular gap in endothelial cells, thereby increasing the permeability of the BBB to both small and large-molecule substances (Wang K. et al., 2023). Here, we focus on reviewing different types of nanomaterials and physical methods for drug delivery through the blood–brain barrier, as well as their physical principles and transport strategies, providing an overview of the latest research findings in this field (Table 1).

**Table 1:** Effective strategies for BBB regulation and crossing.

Strategy	Material/technique	Advantage	Limitation
Para-cellular permeability	Permeation enhancer	Efficiency, low toxicity; Enhanced tight junction permeability	Non-specificity; Cerebral edema; Neurological damage
	Minoxidil sulfate	Inhibition of tight junction protein expression	Side-effects based on drug toxicology
	External stimulus	Reversible openness; Enhanced tight junction permeability	Non-specificity; penetration
Ligand modification	Transferrin	High specificity; Affinity to transferrin receptor	High concentrations of transferrin in the circulatory system
	Low-density lipoprotein	Dual-targeting; Affinity to low-density lipoprotein receptor	Selectivity of binding carriers
	Insulin or anti-insulin receptor	Highly expressed; Affinity to insulin receptor	Metabolic dysfunction affecting selectivity
Membrane coating	Red cell membrane	Long circulation and preventing from immune clearance; Affinity to CD47 proteins receptor	Loss of loading activity
	Macrophage membrane	Bio-compatibility and avoidance behavior; Tumor-associated macrophages regulate tumor progression, invasion, and recurrence	The immune cold tumor state presented by the central nervous system
	Natural killer cell;	High sensitivity; Naturally undergo immunosurveillance	The unique immune system of the brain, the immune cold tumor state presented by the central nervous system
	Exosome	Bio-compatibility and Stability; Biological regulation of source and adjacent cells	Non-specificity

## 2 Strategies for the BBB modulation and penetration

**Figure 1:**
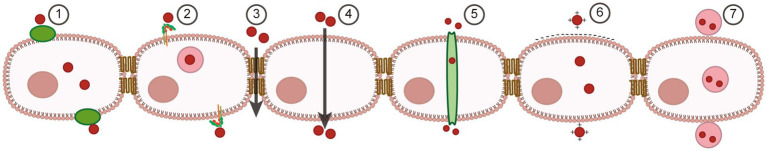
Strategies and materials for BBB regulation and brain-targeted drug delivery. (1) Transporter-mediated transcytosis. (2) Receptor-mediated transcytosis. (3) Para-cellular transportation. (4) Lipophilic pathway. (5) Efflux pump-mediated transcytosis. (6) Adsorptive transcytosis. (7) Vesicle-mediated transcytosis.

The BBB precisely regulates the diffusion and trans-cystic transport of endogenous molecules and impedes the majority (>95%) of exogenous molecules to maintain the homeostasis of the internal environment of the brain, the most complex organ in the human body (Salmina et al., 2021; Zhang et al., 2023a). Therefore, it has been an ongoing concern of researchers to facilitate drug delivery through the BBB to the lesion site. Up to now nanomaterial-based drug delivery systems for penetrating the BBB have consisted of three main components; first, polymers (Canal et al., 2011; Mu et al., 2019; Maghsoudi et al., 2020; Liu et al., 2021), metal nanoparticles (Mortezaee et al., 2019; Kuchur et al., 2020), carbon nanotubes (Lanone et al., 2013; Raphey et al., 2019) and other nanomaterials combined with applied stimuli opened the light for penetrating the BBB; subsequently, liposome-based nanoparticles further improved penetration (Cheng et al., 2021; Tenchov et al., 2021; Wang Y. et al., 2023); and in recent years, biologically based, application of multi-layer technologies combining chemistry and physics, such as biomimetic nanomembranes, have constructed more flexible and stable nanoparticle drug carriers, enabling precise control and release of drugs *in vivo* (Turchanin and Golzhauser, 2016; Jaksic and Jaksic, 2020; Mohammed-Sadhakathullah et al., 2023). A number of brain-targeted therapeutic strategies based on nanomaterials are currently in different clinical trials (Kumthekar et al., 2021; Gusdon et al., 2022). In the following, we will describe the current status and prospects of nanomaterials based on different transportation mechanisms across the BBB (Figure 1).

### 2.1 Para-cellular transport

Cellular para-cellular transport is unmediated and passively reduces the concentration gradient, or it is driven by the osmotic pressure (for water) or solvent resistance of the solute (Almutairi et al., 2016; Xu L. et al., 2019; Qi D. et al., 2023). Because there are no transporter proteins that can be saturated, cellular bypass transport also has the advantage of matching uptake rate to load. Although the tight junctions of the cell gap restrict para-cellular transport of ions, polar molecules, and most macromolecules, a number of small, soluble molecules can nevertheless travel into the brain via this pathway. One way to enhance para-cellular drug transport is to use active excipients that modulate TJs, such as permeation enhancers (PEs), including methanol, silver-leaf lactone, and others (Brunner et al., 2021; Mojarad-Jabali et al., 2021). These compounds target TJs through non-specific interactions to open the para-cellular cleft and increase the transport of small molecules. Streptococcus is an important pathogen causing bacterial meningitis, and a large number of studies have shown that the pathogenic bacteria adhere and gather on the endothelial cells of cerebral micro-vessels, and by destroying the tight junction proteins between the endothelial cells, they cause a change in the permeability of the blood–brain barrier, break through the blood–brain barrier, and enter the central nervous system, In addition, other bacteria that cause meningitis, such as *Escherichia coli*, are also capable of increasing the permeability of the BBB (Al-Obaidi and Desa, 2018; Lei et al., 2023; Mi et al., 2023; Villalba et al., 2023; Yang R. et al., 2023; Yang et al., 2024a). Accordingly, researchers have developed pathogen-associated derivatives that bind to claudins to open para-cellular clefts and increase the penetration of small compounds. Due to the lack of in-depth *in vivo* biosafety and efficacy studies, these active excipients have rarely reached clinical trials. Another therapeutic strategy utilizing para-cellular transport is to reduce the expression of tight junction proteins. MS, an adenosine 5′-triphosphate-sensitive potassium channel (KATP channel) activator, time-dependently inhibits occludin and claudin-5 expression by modulating the reactive oxygen species (ROS)/RhoA/PI3K/PKB pathway, increases tight junction permeability, and facilitates drug transport (Gupta et al., 2022). Han et al. (2016) developed auto-catalytic brain tumor-targeting poly (amine-co-ester) terpolymer nanoparticles (ABTT NPs) co-coated with three blood–brain barrier modulators, Lexiscan, NECA, and minoxidil, which induced the aggregation of nanomaterials in the region of the glioma through the down-regulation of the expression of tight junction proteins and mediated the gene therapy and chemotherapy of brain tumors.

### 2.2 Trans-cellular transport

Trans-cellular transport is the process by which bio-molecules in the peripheral circulation are taken up from one side of the endothelial cell across the plasma membrane into the cell with the aid of receptors (RMT), carriers (CMT), or direct adsorption-mediated cytotranspiration, and are subsequently delivered across the cytoplasm from the other side to the brain (Armulik et al., 2010; Andreone et al., 2017; Fung et al., 2018). Among these processes, brain-targeted delivery systems based on RMT to realize pharmaceuticals have been the most extensively researched (Patel and Patel, 2017; Stocki et al., 2021).

Following this line of thought, many targets that are highly expressed on endothelial cells of the blood–brain barrier have been identified. The transferrin receptor is widely expressed on endothelial cells of the BBB and mediates the brain transport of transferrin-iron complexes to maintain the homeostasis of the internal environment of the CNS. Meanwhile, the vigorous metabolism of most brain tumors and the increase in iron demand leads to the elevated expression of the transferrin receptor on the surface of the tumor cells, which is about 100 times that of the normal neuron, so that, by using the transferrin receptor-ligand binding effect, dual brain-targeted delivery can be realized (Kawabata, 2019; Sahtoe et al., 2021). Currently, researchers have identified and developed a variety of transferrin receptor-targeting ligands for modifying the surface of nanomaterials, such as transferrin, targeting peptides, corresponding antibodies, and their fragments (Shen et al., 2019; Koneru et al., 2021; Li J. et al., 2023). Ag Seleci et al. (2021) encapsulated magnetic nanoparticles within liposomes with surface-modified transferrin, and demonstrated that the modified transferrin significantly increased the enrichment of brain tumor regions, as well as markedly enhanced the uptake of the nanomaterials by tumor cells under magnetic guidance. Prades et al. (2012) made fusion peptide-functionalized gold nanoparticles (5-13 nm) that were able to attach TfR and β-amyloid. This nanoparticle was also detectable in neuronal cells in the cerebral cortex after intraperitoneal injection into mice. Although other research groups have demonstrated that small-sized gold nanoparticles can access the brain without any target modification, the introduction of the targeted ligand also significantly improved the efficiency of transport.

The low-density lipoprotein receptor (LDLR) family are membrane-mosaic proteins that mediate the cytosis of low-density lipoproteins, mainly including LRP1, LRP1B, LRP2, LRP5, LRP8, LDLR, and VLDLR. LDLR is not only a ubiquitously expressed receptor, but it is also extensively expressed in the brain, making it an effective transport protein for therapeutic pharmaceuticals (Li et al., 2001; Defesche, 2004; Gent and Braakman, 2004). Angiopep-2 is a 19-amino acid peptide originating from the Kunitz domain of bovine protein repressor peptidases and binds to LRP1 (Habib and Singh, 2022; Thirumurugan et al., 2022; Zhu et al., 2022). Li B. et al. (2022) applied angiopoietin-2 peptide-modified engineered exosomes to trigger transcytosis of endothelial cell, allowing NPs to traverse the BBB and target GBM cells by recognizing the LRP-1 receptor. In addition, apolipoprotein B, apolipoprotein E, and their derivative peptides have the ability to deliver siRNA and nanoparticle targeting to the brain parenchyma (Ishii, 2019; Alagarsamy et al., 2022; Yang L. G. et al., 2023). Furthermore, while some compounds can facilitate brain-targeted delivery of agents, their specific mechanism of action remains unclear. Tween 80 is a hydrophilic surfactant with powerful cell membrane cleavage causing irritation, hemolysis and sensitization (histamine release). In recent years, researchers have found that Tween 80 can be used as a surfactant coated on carriers such as nanoparticles to facilitate their brain delivery (Azhari et al., 2016; Menberu et al., 2021). Xu et al. (2020) prepared rhynchophylline (RIN) loaded methoxy poly (ethylene glycol)-poly (lactic-co-glycolic acid) (mPEG-PLGA) nanoparticles (NPs) and subsequently added Tween 80 to form T80-NPs-RIN. It was demonstrated that T80-NPs-RIN had a significantly higher rate of transport in a BBB model *in vitro*, with drug accumulation increasing over time and osmotic saturation being reached after 3 h, compared to RIN or NPs-RIN alone.

Of course, there are other types of receptors that mediate transcellular transport of carriers. Insulin receptors on brain capillary endothelial cells have been demonstrated to be useful for brain-targeted drug delivery (Vella et al., 2018; Lawrence, 2021; Choi and Bai, 2023). Insulin binds to two different sites on each subunit of the receptor, cross-linking the two receptors, resulting in a high-affinity. However, direct application of insulin as a ligand to prompt nanoparticles to cross the BBB has a number of disadvantages (Boucher et al., 2010; Scherer et al., 2021; Nagao et al., 2023). Firstly, insulin might cause hypoglycemia, which requires close monitoring of blood glucose changes or early interventions, and in addition, it has been indicated that insulin transport across the BBB is related to the type of insulin receptor, e.g., insulin receptors associated with signaling activation are not involved in its transcellular transport. Therefore, researchers generally utilize glucagon mimetic peptide antibodies and derivatives to overcome the aforementioned difficulties and achieve safe and effective brain-targeted delivery of pharmaceuticals (Ulbrich et al., 2011; Betzer et al., 2017; Tashima, 2020). However, neither paracellular nor transcellular transport is only applicable to small molecules of hydrophilic compounds or highly hydrophobic compounds less than 400-600 Da. If high molecular drugs need to be delivered by either method, a transient reversible disruption of the BBB or an increase in local blood–brain barrier permeability is required.

In addition to receptor-mediated transcellular trafficking, transporter (carrier) and adsorption-mediated brain-targeted delivery of medications has been intensively investigated for applications (Persidsky et al., 2006; Pardridge, 2015; Balzer et al., 2022; Ni et al., 2022). In the first place, glucose transporters and glutathione transporters are the two most commonly utilized transporter proteins that facilitate nanoparticle penetration of the BBB for the delivery and aggregation of pharmaceuticals. Glucose transporters (e.g., GLUT1) are highly expressed on brain capillary endothelial cells and various tumor cells, including brain tumors, while glucose is a principal source of energy for brain and tumor hypermetabolism; thus, surface-loaded glucose ligands enable sequential dual-targeted delivery of therapeutic agents (Abbott, 2002; Serrano et al., 2012). For example, Jiang et al. developed 2-deoxy-D-glucose modified poly (ethylene glycol)-co-poly (tri-methylene carbonate) nano-system (D-Glu-NP) with glucose as a ligand, realizing the above-mentioned dual-targeting strategy of agents (Jiang et al., 2014). Glutathione, as an endogenous antioxidant, is present in almost all cells and can contribute to the maintenance of normal immune system function and has antioxidant effects and integrative detoxification (Meister, 1991; Cooper and Kristal, 1997). Studies have demonstrated that glutathione is significantly higher in the brain than in the peripheral circulation or other tissues, and glutathione transporters are efficiently expressed on brain capillary endothelial cells, which exert an influential role in the maintenance of CNS homeostasis. Recently brain-targeted delivery systems for glutathione transporters have been gradually developed. In addition, adsorption-mediated transcellular transport has been extensively explored for brain-targeted drug delivery (Scherrmann, 2002; Zhang et al., 2023b). Brain endothelial cell membranes are naturally negatively charged, and transcellular transport is achieved by electrostatic interactions with positively charged agents or pharmaceutical carriers. Cell-penetrating peptides (CPPs) are short, positively charged peptides (< 20 amino acids) that promote cellular uptake and absorption of molecules ranging from nanoparticles to small compounds to large DNA fragments, a typical adsorption-mediated transcellular transport (Guidotti et al., 2017; Zhou et al., 2022; Zorko et al., 2022). Since CPP-mediated drug delivery lacks cell specificity and targeting, it is usually combined with corresponding targeting peptides to realize the dual effects of drug crossing the BBB and focal aggregation. Zhong et al. developed co-functional tandem nano-compartments, termed ANG20/TAT10-Ms, loaded with both Angiopep-2 and the transcription factor TAT (a CPP), and found that ANG20/TAT10-Ms had significantly higher glioma accumulation rates compared to nanoclusters that included only Angiopep-2 modifications in the U87MG glioma mouse model.

## 3 Penetration strategies for the BBB

### 3.1 Membrane camouflage for brain targeting

**Figure 2:**
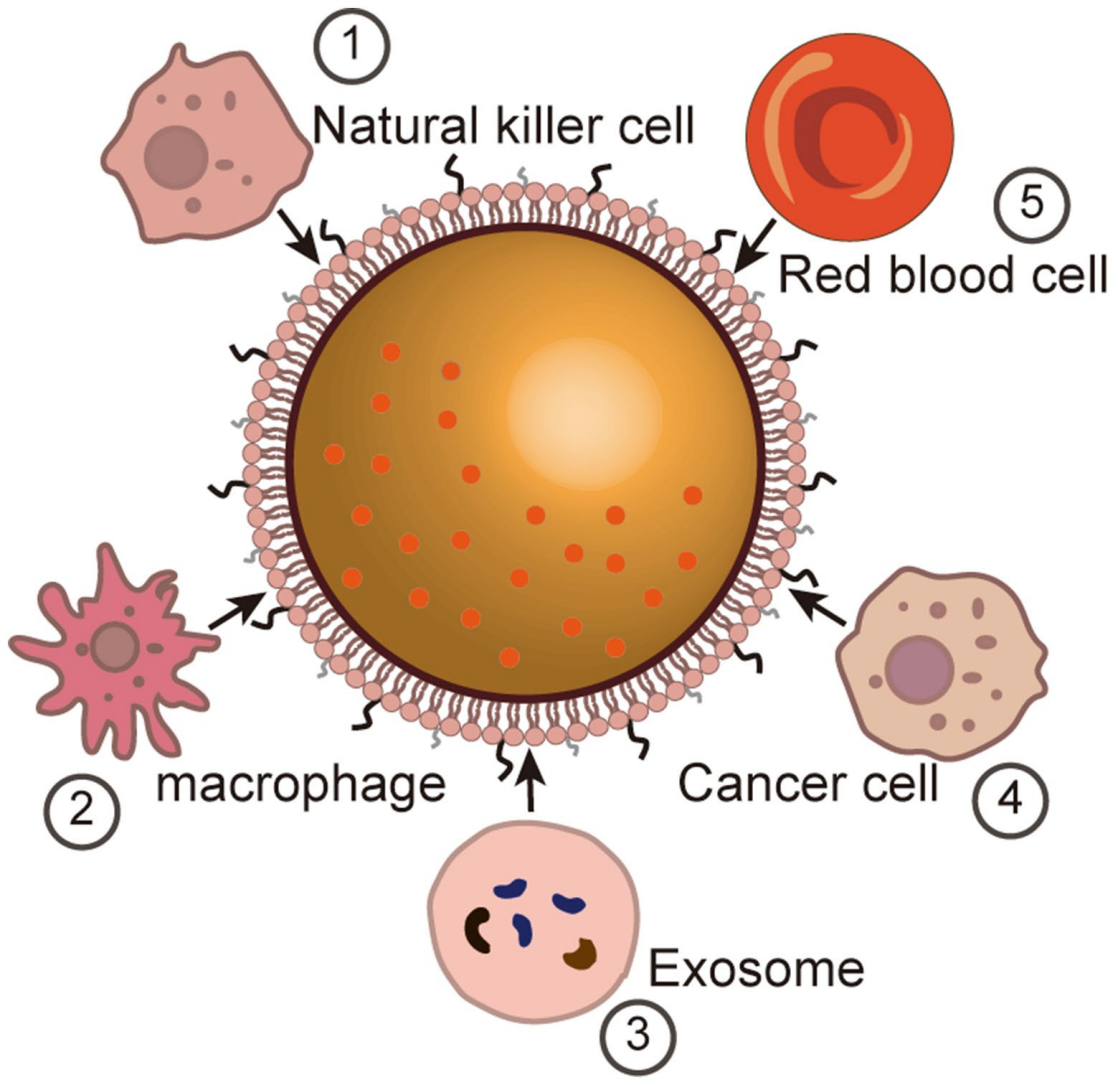
Membrane camouflage for brain targeting. Immune cell-based membrane camouflage (1) Natural killer cell (NK). (2) Macrophage. Vesicle-based membrane camouflage (3) Exosome. (4) Cancer cell-based membrane camouflage. (5) Red blood cell-based membrane camouflage.

In the past decades, nanotechnology has achieved promising results in biomedical applications in a variety of directions, including therapeutic, diagnostic, etc. However, the inherent rejection of the immune system prevents some, if not the majority, of nanoparticles that enter the body from functioning (Mundt et al., 2022; Salvador and Kipnis, 2022; Xu et al., 2022). In order to avoid detection and removal by the immune system, researchers have attempted to evade capture by the immune system through methods such as particle surface modification (Yang et al., 2018; Cao et al., 2020; Li Y. et al., 2023). For example, polyethylene glycol (PEG) coated on the surface of nanoparticles can comprise a kind of “invisible coating” (Fang et al., 2023). This PEGylation has proven to be effective in prolonging the half-life of most nanoparticles, however, acquired immunity is still able to accelerate clearly with more frequent administration. Furthermore, in order to improve the focal targeting and specificity of nanoparticles, a great deal of work has focused on the identification, characterization and production of specific ligands. As the construction of nanocarriers becomes progressively more complex, the process of ligand modification inevitably becomes difficult to control. In recent years, the modification of nanoparticles utilizing natural membrane materials such as cell membranes and exosomes has become a new area of research interest. The interaction of these (biomimetic) nanoparticles with cell membrane-derived platforms allows them to avoid clearance by the immune system and, at the same time, has natural targeting properties that enable them to travel through complex biological environments to reach and accumulate in focal areas (Figure 2).

#### 3.1.1 Brain targeting based on cell membrane encapsulation

Erythrocytes have natural biophysical properties and stability that enable them to be excellent “shells” for safe and immune-compatible drug delivery systems (Cui et al., 2020; Zhang et al., 2022). In addition, erythrocyte membranes are readily available, simple to prepare, and uniform in size and shape. In order to improve the efficacy of drugs for the treatment of ischemic stroke, Lv et al. (2018) developed a bioengineered ROS-responsive nanocarrier for specific brain-targeted delivery of the neuroprotectant NR2B9C to reduce the extent of ischemia-induced brain injury by targeting the ROS upregulation in ischemic neurons. This nanocarrier consists of a dextran polymer core modified with ROS-responsive borate and a red blood cell (RBC) membrane shell inserted with stroke homing peptide (SHp) (Lv et al., 2018). Thus, this nanoparticle (SHp-RBC-NP) controls the release of NR2B9C upon entry into ischemic brain tissues triggered by high ROS. The potential of SHp-RBC-NP for the treatment of ischemic stroke was systematically evaluated *in vitro* and a rat middle cerebral artery occlusion (MCAO) model. The results of *in vitro* experiments showed that SHp-RBC-NP was well protected against glutamate-induced cytotoxicity. *In vivo* pharmacokinetic (PK) and pharmacodynamic (PD) assays also further demonstrated that the nanoparticles significantly prolonged the systemic circulation time of NR2B9C, enhanced the active targeting of ischemic regions and attenuated ischemic brain damage in MCAO rats.

Leukocytes can cross the BBB and reach the lesion area in the brain, including macrophages, dendritic cells (DCs), and natural killer (NK) cells. Therefore, immune cell membranes are favorable membrane-encapsulated materials because of their excellent biocompatibility and the adverse effect of non-recognition of normal cells (Artyomov et al., 2016; Crinier et al., 2020; Hilligan and Ronchese, 2020; Kameritsch and Renkawitz, 2020; Wu et al., 2020; Anderson et al., 2021; Kadomoto et al., 2021; Lee et al., 2021; Wen et al., 2022; Wiernicki et al., 2022; Chen X. et al., 2022; Galati and Zanotta, 2023).

Naturally occurring molecules on the surface of macrophage membranes can confer a number of properties to encapsulated nanoparticles, including the BBB penetration, brain tissue targeting, and immune evasion. For example, C-C chemokine receptor 2 (CCR2) (Hajal et al., 2021; Quaranta et al., 2023), and intercellular adhesion molecule-1 (ICAM-1) (Dietrich, 2002; Soldati et al., 2023) can direct macrophage membrane-encapsulated nanoparticles to the tumor region, and in addition, α4 and β1 integrins on the macrophage membrane can interact with vascular cell adhesion molecule-1 (VCAM-1) on the membrane of the cancer cells, allowing macrophage membrane-encapsulated nanoparticles to be targeted to the cancer cells and cancer metastasis (Rosenman et al., 1995; Haarmann et al., 2015; Cheng V. W. T. et al., 2019). Jiang et al. constructed nanoparticles loaded with chemotherapeutic drugs and encapsulated using macrophage membranes (cskc-PPiP/PTX@Ma) (Zhang et al., 2018). It was observed that the nanoparticles crossed the BBB with the assistance of various chemokines on the surface of macrophage membranes to reach the tumor region, and were subsequently shed through morphological changes driven by stimulation of the extracellular microenvironment of the tumor. After the nanoparticles are internalized, the loaded drug is rapidly released from the nanoparticles in response to endosomal pH, thereby enhancing the tumor killing effectiveness. This combination of bionic cell membranes and cascade-responsive nanoparticles builds an effective drug delivery system tailored to the tumor microenvironment.

Natural killer cells (NK cells), important immune cells in the organism, are morphologically large granular lymphocytes, and although they lack tumor antigen-specific cell surface receptors, they have a number of alternative receptors that can recognize cancer cells, including NKG2D (Dhar and Wu, 2018; Liu et al., 2019), NKp44, NKp46, and DNAM-1,(Horton and Mathew, 2015; Liu et al., 2019; Focaccetti et al., 2022; Murayama et al., 2022; Cifaldi et al., 2023) which have excellent biocompatibility and tumor targeting properties. Tang and coworkers developed nanorobots by coating an aggregation-induced emission-active polymer endoskeleton with a membrane derived from NK cells to mimic NK cells (Su et al., 2021). Mechanistic studies indicated that receptors from NK cells to the surface of the nanorobots play a primary role in BBB crossing and tumor recognition.

#### 3.1.2 Brain targeting based on exosomes

Exosomes are membranous vesicles released into the extracellular matrix by fusion of intracellular multivesicular bodies (MVB) with the cell membrane (Zhang et al., 2015; Yang et al., 2019; Zhang et al., 2020). Almost all types of cells, which can produce and release exosomes, are nanoscale lipid inclusion structures with a diameter of 30–100 nm. Because of their origin from parental cells, they have excellent biocompatibility and tissue-organ targeting properties (He et al., 2018; Kalluri and LeBleu, 2020; Ocansey et al., 2020). Therefore, changing the cellular origin of exosomes may be an effective strategy to induce brain-targeted delivery (Wu et al., 2022; Zhan et al., 2022; Kim et al., 2023; Tao et al., 2023; Yang et al., 2024b). In a mouse stroke model, neural stem cell-derived EVs showed enhanced CNS delivery efficiency compared with MSC-derived EVs. However, it was discovered that exosomes from different cell types without any modification had a delivery efficiency of <1% to the brain after systemic injection, implying that exosomes have a natural tendency to ignore the BBB (Zhang and Yu, 2019; Peng et al., 2020; Liang et al., 2021). As a result, researchers have attempted various modifications to the exosome surface to improve its brain-targeting ability, such as the aforementioned receptor-mediated transcellular transporter action. Kim et al. utilized transferrin receptor (TfR)-modified exosomes to construct TfR-exo, and by coupling the T7 peptide to Lamp2b, compared to the unmodified exosome, TfR-exo had favorable BBB penetration and glioma targeting (Kim et al., 2020; Choi et al., 2022).

### 3.2 External stimuli mediate transient opening of the partial BBB

**Figure 3:**
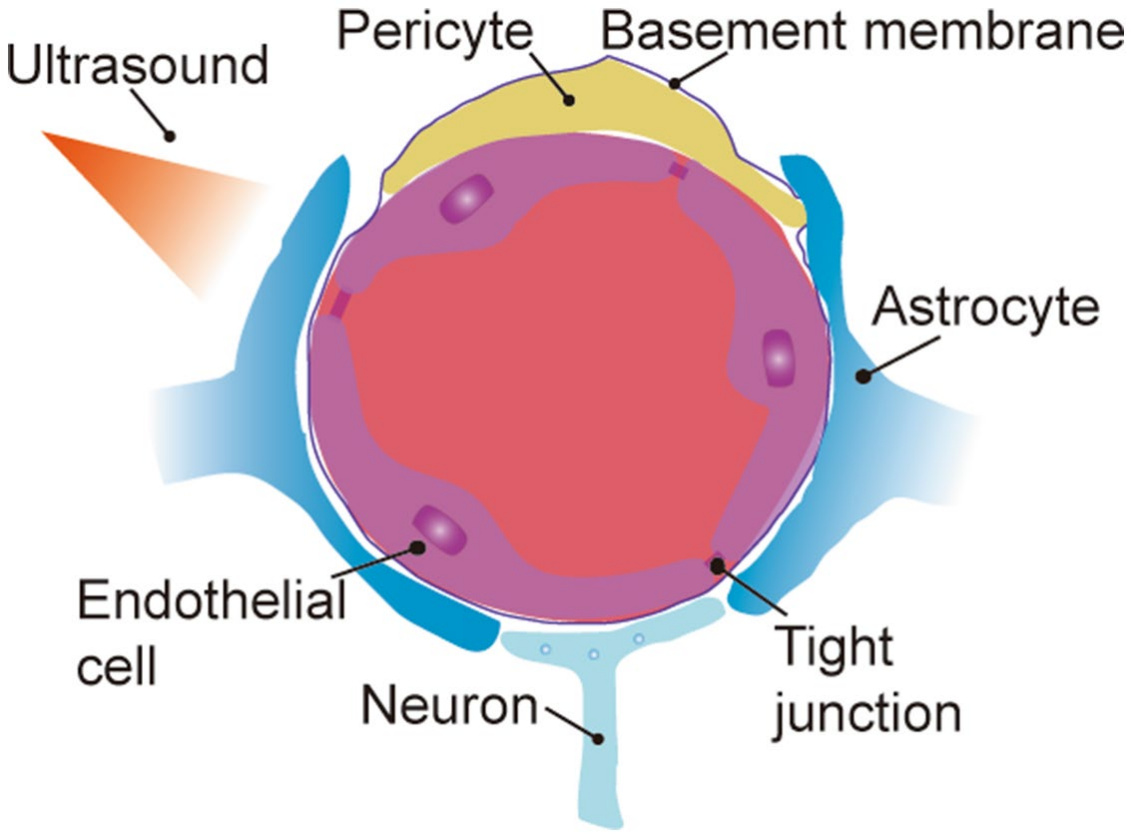
External stimuli mediate transient opening of the partial BBB. (1) Focused ultrasound combined with micro-bubbles. (2) Surface acoustic wave.

Many chemotherapeutic drugs are unable to achieve transcellular brain-targeted delivery only through the aforementioned transmembrane transport pathway due to their large molecular weights. Therefore, transient opening of the local BBB through external stimulation with the assistance of various energy-converting materials and modulation of its permeability is an effective strategy to promote brain-targeted delivery of drugs (De Cock et al., 2015, 2016; Mulik et al., 2016; Szablowski et al., 2019; Cheng B. et al., 2019; Chen J. et al., 2022) (Figure 3).

Focused ultrasound (FUS) combined with microbubbles (MBs) is used extensively in the clinic for enhanced angiography of well perfused organs such as the heart, liver, and kidneys (Wang et al., 2022). In recent years, this technique has been applied to open the BBB for targeted brain delivery of medications (Kamimura et al., 2019; Vince et al., 2021; Yang et al., 2021). In recent years, the US FDA has approved monoclonal antibody drugs for the treatment of Alzheimer’s disease (AD). Relevant studies have shown that focused ultrasound could effectively improve the concentration of monoclonal antibodies in the brain and enhance the therapeutic effect of AD (Ma et al., 2023). The principle of this technique is that low-frequency focused ultrasound is not restricted by the skull, and after penetration, it can gather in the brain and lead to a series of cavitation effects in microbubbles in intracranial blood vessels, which stretches the structure of the BBB and ultimately induces an increase in the permeability of the blood–brain barrier (Hijnen et al., 2017; Nowicki, 2020). The BBB can be briefly and reversibly opened by FUS with the assistance of MBs to concentrate the ultrasound energy on the localized area of the BBB. How to precisely control the FUS energy to keep the opening of the BBB within a safe and effective range is a question that researchers need to consider (De Cock et al., 2016; Sun et al., 2017; Xu S. et al., 2019). Fortunately, focused ultrasound combined with microbubble-mediated BBB opening has entered clinical trials in recent years, and its safety and efficacy have been supported by clinical evidence (Rezai et al., 2020; Mehta et al., 2023). The main application limitation of bubbles as FUS-mediated BBB opening response is the bubble size. Chen et al. (2019) synthesized a porous lipid-polymer hybrid MBs (lipid/PLGA MBs). These MBs can encapsulate drugs in the inner lumen and simultaneously open the local BBB by FUS-triggered cavitation effect to release the encapsulated medications at the lesion site; however, these MBs have micrometer-sized particles, resulting in ultrasound-perforating effects only within the blood vessels (Chen et al., 2019). Studies have found that nanoscale bubbles, including nanobubbles, nanodroplets, echo-exosomes, and liposomes, as well as air bubbles, are highly stable and that binding to the FUS more facilitates the transport of biomolecules across the cellular and tissue barriers, even though the reduction in the size of the bubbles results in a hardening of their shells and a reduction in the echo signal (Kim et al., 2015; Suarez Escudero et al., 2018).

Surface acoustic wave (SAW) is an acoustic wave that propagates along the surface of a medium with excellent properties such as concentrated energy and easy manipulation, which makes its application in biomedical fields gradually widespread (Wang K. et al., 2023). It has been discovered that SAW stimulation can influence the distribution of tight junction proteins between endothelial cells in brain capillaries without affecting the expression of tight junction proteins or the activity of endothelial cells, and enlarge the cellular gaps between endothelial cells, thus facilitating macromolecules to penetrate the BBB and enter into the brain (Steed et al., 2010; Ding et al., 2013; Guo et al., 2021; Peng D. et al., 2021; Kim et al., 2022).

## 4 Conclusion and perspectives

The BBB serves as a natural barrier to maintain homeostasis of the internal environment of the CNS. However, this barrier is a double-edged sword that protects the brain from toxins and pathogenic bacteria, but the endothelial cels and the tight junctions between them also impede the brain permeability of most therapeutic drugs which largely affects their therapeutic efficacy for CNS disorders (Iovino et al., 2016; Peng D. et al., 2021). With the rapid development of nanotechnology and the in-depth integration of medical engineering crossover, it brings light to the regulation of blood–brain barrier permeability and targeted drug delivery. Researchers have designed and fabricated many drug-targeting delivery systems with unique physicochemical properties and multifunctional nano-substrates according to the demand to achieve drug brain focal aggregation by modulating various transcellular transport pathways and BBB permeability (Qian et al., 2020). This paper summarizes the research progress of various drug delivery systems in recent years and the problems that require attention/solution. Nanomaterials-based delivery systems have excellent bio-compatibility for drug delivery through the molecular transport pathway of the BBB without causing damage to normal organs in the human body, especially in recent years with the continuous advancement of nanotechnology, which has been conjugated to biological membranes or linked to relevant antibodies to enable them to acquire complex therapeutic functions. Liu et al. (2023) developed engineered artificial vesicles (EAVs), ANG-TRP-PK1@EAVs, which have similar properties to secreted exosomes but with higher yields. ANG-TRP-PK1@EAVs had efficient BBB penetration and GBM targeting ability. Adriamycin-loaded EAVs (ANG-TRP-PK1@DOX) have not altered the properties of EAVs, which can penetrate the BBB, reach the GBM, and kill tumor cells in the *in situ* GBM mouse model. Researchers generally increase the aggregation penetration of nanoparticles in the BBB by ligand coupling, but intravenous injection leads to redirection to other organs such as the liver, so altering the route of administration to increase BBB aggregation is a pressing issue, such as nasal administration and convection-enhanced administration (Yang et al., 2022; Pickering et al., 2023). Physical methods such as FUS achieve reversible brain-targeted drug delivery by briefly opening the local blood–brain barrier through low energy without serious complications or side effects. For example, MR-guided focused ultrasound (MRgFUS) combined with intravenous microbubble drug delivery has been applied to open the focal temporary BBB in patients with neurodegenerative diseases and brain tumors, and it has become a therapeutic tool for drug delivery in neurorecovery therapy (Gasca-Salas et al., 2021). However, this method is unable to realize the precise delivery of drugs. In recent years, nanodelivery systems combined with physical methods have become a new option, such as the FUS/microbubble-assisted BBB opening for intravesical delivery of lipid nanoparticles encapsulating mRNA to the brain studied by Ogawa et al. (2022). The intensity of the FUS irradiation was optimized to 1.5 kW/cm2 without hemorrhage or edema (Ogawa et al., 2022). In conclusion, the delivery methods described in this paper each have their own advantages, and intensive cross-fertilization will further improve the delivery efficiency.

Despite the tremendous achievements of this engineering technology in the field of brain-targeted delivery, and even some nano-delivery platforms have progressed to the clinical trial stage, there are still technical difficulties or problems in this technology that require researchers to overcome and optimize. (1) Peripheral circulation time of nanoplatforms (Ohta et al., 2020). It was discovered that the physical properties of nanomaterials, including size, charge, and composition, affect blood circulation time. This not only reduces the efficiency of brain-targeted drug delivery, but also might cause accumulation of materials in peripheral tissues and organs, which raises biosafety concerns. In addition, the peripheral immune system may phagocytose and destroy the BBB, reducing its serum half-life. Researchers generally make nanomaterials functional for immune escape through ligands and membrane camouflage, in addition, polyethylene glycolization is commonly applied to prolong the half-life of nanomaterials and avoid their destruction. In conclusion, during the construction of nanomedicine delivery platforms, it is essential to consider how to make them rapidly aggregate in the lesion area. (2) Spatio-temporal deliberate targeting. After nanomaterials have been modified or successfully penetrated the BBB with the assistance of their own physicochemical properties, they will not be completely pooled in the focal area, and other therapeutic modalities other than drug delivery with the help of nanomaterials, such as thermotherapy and catalytic therapy, require precise control of their temporal and spatial distribution, thus realizing precise treatment and reducing the damage to the normal brain tissues. (3) Long-term biosafety concerns. Favorable biosafety is an issue that must be considered and resolved for nanomedicine delivery platforms to truly achieve clinical applications. Although most of the literature findings indicated that nanomaterials do not inflict acute toxic damage to cells, tissues, and organs, their long-term distribution and metabolism should be further evaluated. Studies have identified that some nanomaterials may have excellent biocompatibility, pharmacological and toxicological evaluations have revealed that these materials may reside in areas of normal brain tissue and cause long-term brain damage. Therefore, in addition to exploring the molecular mechanisms of nanomaterials to penetrate the BBB and disease treatment, long-term biosafety assessment is also an issue that researchers must pay attention to. (4) homogeneity and reproducibility. Subtle changes in the physical and chemical properties of nanomaterials, such as size and shape, can influence their functions; in addition, the reproducibility of penetration, targeting and other functions is also a direction that researchers need to focus on. This puts higher demands on the preparation techniques and processes of nanomaterials. Although this technology is still facing problems that require urgent solutions, in view of the favorable results achieved so far, we believe that further in-depth cross-development of medical engineering technologies will provide a broader platform for precise BBB penetration and brain-targeted therapies.
